# Calculous Pyelitis*Specially Reported for the Buffalo Medical and Surgical Journal.

**Published:** 1883-01

**Authors:** William Pepper

**Affiliations:** Professor of Clinical Medicine in the University of Pennsylvania


### BUFFALO

### Medical and Surgical Journal.

VOL. XXII. JANUARY, 1883. No. 6.

### Original Communications.

### CALCULOUS PYELITIS.*

A CLINICAL LECTURE DELIVERED AT THE HOSPITAL OF THE UNIVERSITY OF PENNSYLVANIA, DEC. 2, 1882.

BY WILLIAM PEPPER, M. D., L.L. D.,
Professor of Clinical Medicine in the University of Pennsylvania.

GENTLEMEN—You may remember that I brought before you, a few weeks ago, a man exhibiting the following symptoms: He was a middle-aged man, and had complained of frequent micturition and occasional attacks of pain in the left side extending towards the left groin, but without any symptoms of stone in the bladder. The urine, on standing, deposited a small amount of pus which was easily diffused. There was but little mucus. Examination with the microscope showed pus corpuscles and phosphates, but no tube-casts. The urine also contained albumen, but no more than could be explained by the amount of pus present. There was but little tenderness on pressure over the kidney, and he stated distinctly that jars and jolts to his body did not cause pain in the spot to which he referred his attacks of pain. This case seemed, as I then stated

*Specially Reported for the BUFFALO MEDICAL AND SURGICAL JOURNAL.

to you, to be one of calculous pyelitis, that is, inflammation of the pelvis of the kidney dependent upon the existence there of a calculus.

I pointed out to you that the symptoms of calculous pyelitis varied somewhat, according as the calculus remained in or had been expelled from the kidney; and secondly, as the calculus was large, irregular and very free to move and thus press upon and irritate the lining membrane, or as it was smooth or had become encysted so that it was almost like an inert body, and the inflammation which it had excited remained.

Different cases differ greatly in regard to the amount of pain. The pain is of two kinds. Sometimes there are only spells of renal colic, by which we mean sudden attacks of pain which appear in the region of one or the other kidneys, extending around under the short ribs, over the crest of the ilium, down into the groin, often associated with retraction of the testicle and pain at the end of the penis. These spells last a varying number of hours and vary much in severity, being sometimes a pretty bad pain, at other times being so severe that the patient is in a state of collapse, with cold extremities, pinched face, vomiting and agony, requiring the use of anæsthetics, or the hypodermic injection of morphia in order to afford any relief. In addition to these attacks of pain, there is a greater or less degree of tenderness on pressure and pain on shaking or jarring the body. Sometimes the tenderness on pressure is extreme, while at other times the deepest pressure will cause but little suffering. Not every attack of renal colic is attended with the passage of a calculus. It is due at times to a neuralgic paroxysm affecting the kidney, attended by a sensitive state of the organ, some congestion and a little derangement of the urine, or occasionally with the passage of fine urinary gravel. The attack of colic may be attended by the passage of a minute calculus. The pain is experienced while the stone is passing through the ureter. When it escapes into the bladder the patient may experience instant relief. Perhaps two or three days afterwards, while urinating, he will hear something drop into the vessel, and on examining, he finds a minute calculus as large as a spring pea. Sometimes there will be an impediment in the urethra, but after a number of strong efforts a little calculus is passed. Attacks of colic may also be due to the presence in the pelvis of the kidney of a stone too large to pass through the ureter, but which occasionally stops up the orifice of the ureter, preventing the escape of the urine and causing spasm and pain which last for an indefinite time, and then as the stone shifts its position, the pain disappears, the urine escapes and the spell is over. The tenderness and pain on motion are dependent partly upon the inflammation of the lining of the pelvis and irritation of the kidney structure, and in some cases partly upon the presence of a calculus which can be moved and jarred, thus wounding the sensitive structures; according as there is or is not a calculus, according as the inflammation is acute or chronic, will be the amount of tenderness on pressure, and of pain on agitation.

Another symptom, which varies much in different cases, is hæmaturia, that is, the passage of blood from the penis. This is a common symptom. It occurs in disease of the lining of the bladder and of the neck of the bladder far more frequently than in disease of any other part, but it also takes place in connection with renal calculus. If the hæmaturia appears in connection with symptoms referable to the bladder, the blood probably comes from its lining or the vessels at the neck. If, on the other hand, the hæmaturia occurs in a person who has had renal colic, fixed pain and soreness over the region of the kidney, and especially if before the attack of bleeding he has a spell of pain, the hemorrhage is probably due to renal irritation. There are some differences in the appearance of the blood, as it comes from one or the other of these two localities. If it comes from the bladder, it may be of a darker red than when it comes from the kidney, or it may be greater in quantity, and form clots which will be firm and of a dark color, or the blood may be mixed with a great deal of vesical mucus. If the blood comes from the kidney, it is usually readily diffused through the urine by agitation; when examined under the microscope, the blood corpuscles are found separate and distinct, and they indicate by their crenated, shriveled look, that they have been lying in the urine for some time. Clots are rare. At times, if the substance of the kidney is involved, there may be a slight hemorrhage from the tubules, which will appear as little tube-casts.

The urine presents other characteristics besides those due to the presence of blood. The urine in renal calculus, if pyelitis has been established, is always albuminous, the amount of albumen being always small and directly proportional to the amount of pus thrown down in the sediment; so that we can, by experience, estimate from the amount of pus in the sediment the amount of albumen in the super-natant liquid. In disease of the bladder, where there may be a great deal of mucus, the urine is not of necessity albuminous; but if the inflammation is severe, with the formation of pus, the urine in cystitis is albuminous, but the amount of albumen is not proportional to the amount of sediment, because there is present a large quantity of mucus, which is not necessarily associated with the presence of albumen. In organic disease of the kidney, the amount of albumen bears a much larger proportion to the amount of sediment than it does in either of the other two affections.

The epithelial cells found in the urine of cystitis and in that of pyelitis differ in character. In the former disease, they are of the squamous variety; in the latter they have more of a spheroidal shape, showing that they come from the higher parts of the urinary passages.

Frequency of micturition is a common symptom in all these affections, that is, cystitis, pyelitis and Bright's disease. This differs so much in different patients that we can not attach to it any material diagnostic value.

Lastly, these patients are anæmic; show the effects of their suffering; have disturbed sleep and usually show loss of flesh.

Having refreshed your memories upon the points to which I alluded on the previous occasion, let me refer to the case which has induced me to again speak of this subject.

This man has, for some time, been under the care of Dr. J. H. Musser, in the dispensary. He is 58 years old, a laborer by occupation. Thirty-four years ago, he had a severe attack of pain over the region of the left kidney, which passed around to the front but not into the testicle; the pain was not at that time accompanied by bleeding. It came on in spells of a few hours' duration for two or three days; he then returned to his work and felt as well as ever. Nine years later, that is twenty-five years ago, he had, after exposure to the weather, an attack of hemorrhage from the penis, which, at that time, was called hemorrhage from the kidney. Of course, after so long a time has elapsed, it is difficult to say whence the blood came. It may have come from the bladder or from the kidney, more probably from the former. Exposure to cold and damp may produce a sudden congestion of the kidney, which will relieve itself by bleeding; there will be hæmaturia for one or two days, and the patient will then recover without any nephritis being established. Far more frequently, however, such attacks of mild, transient bleeding are from the bladder. The frequency with which hæmaturia occurs from the bladder is very great. It is important that we should recollect this, in order that we shall not be unnecessarily alarmed when we meet with such cases. There is a state of hemorrhoids of the bladder (I cannot call it by any other name), a varicose enlargement of the vessels at the neck of the bladder or at the bas-fond. This is strictly analogous to the state of varicose distention of the hemorrhoidal plexus; and, indeed, the vesical plexus is a part of the hemorrhoidal plexus, and subject to the same causes of distention. I have seen, on several occasions, patients, who had their piles ligated, take to bleeding from the bladder. I have had patients who have had hæmaturia for thirty years, and longer, some of them quite frequently, in whom

I could never find any organic disease of the bladder or evidences of calculous disease, and in whom there was a fair maintenance of general health. The bleeding could only be explained by congestion of the vessels at the neck of the bladder. In such persons, exposure to damp and cold will be apt to be followed by hemorrhage. This was probably the case in this man. There was but one attack of bleeding at that time, and it was unaccompanied by pain and swelling over the bladder. This is a pretty strong indication of a predisposition to irritation, congestion and hemorrhage from the urinary tract. He did not have another spell of bleeding until last spring, seven months ago. He has had, for some years, more or less dyspepsia. This very often attends the diathesis, called the uric acid diathesis. It is in such patients that we frequently find associated urinary calculus. Seven months ago, he had a spell of pain, with bleeding. The hemorrhage has occurred four times since then. There is a very slight increase in the frequency of micturition, and he has to empty his bladder once or twice during the night. This is not at all uncommon when a man has reached the age of 65; and even at 58 there may be a little enlargement of the prostate, causing a slight increase in the frequency of urination. Examination of the urine with the microscope shows the presence of pus cells, epithelium from the higher parts of the urinary passages and blood corpuscles, but very little mucus. He states that there has been some small clots passed; these may have come from the bladder or from unusually free bleeding from the ureter.

During the past seven months he has had a number of attacks of pain, which commence over the left kidney, running down towards the bladder, and are attended with increased frequency of micturition. The spells last for a few hours and are sometimes so severe as to cause nausea and vomiting. He has never passed gravel or a stone. When I press in the region of the left kidney, it "stooms," as he describes it. Sudden jars cause pain in this region. When you meet with a case with such a history, frequent hæmaturia, repeated spells of renal colic, local tenderness, pain and soreness on jarring of the body, and some increase in the frequency of urination, there can be little doubt that you have to do with a case of renal calculus, not merely pyelitis, but also the presence of a calculus. This man probably has a calculus with a uric acid centre and phosphatic concretions in the left kidney, and the pyelitis has been induced by it. The stone being free to move, changes its position at times, causing severe attacks of pain. This case is a fit associate for the milder one which I brought before you some weeks ago. It is an accurate picture of a bad case of calculous pyelitis.

The treatment of these cases is unsatisfactory, tedious and often disappointing. It is not necessary to say that the first thing to be done is to relieve the dyspepsia. It is that which has caused the trouble. The existence of acid dyspepsia, the acid state of the secretions, and the tendency to what is termed the acid diathesis, has led gradually to the formation of minute concretions of uric acid crystals in the calyces or pelvis of the kidney. These have excited irritation, set up inflammation, and thus there has been a precipitation of the salts of the urine, which, mixing with the mucus, have coated the calculus so that it has now grown so large that it cannot escape. Sometimes there is one stone, at other times there are many. They are sometimes found in the tubules, and they may even be found in the body of the kidney. Occasionally they are fastened to the mucous membrane by a mass of lymph. Again, you may find present one or two large movable stones, with prolongations, enabling them to engage in the ureter. All these different conditions are met with. It is impossible, in any given case, to say what is the exact character of the calculus.

As I have said, the first thing to be done is to cure the acid dyspepsia. Note, at the same time, that by curing the acid dyspepsia we render the urine neutral and lessen the irritability of the urinary passages, for acid urine, flowing over these inflamed surfaces, will predispose much more to pain than will the presence of neutral urine. We must also try to prevent further concretions and relieve the irritability of the urinary passages.

Dietetics, of course, stand first in the treatment of the dyspepsia. Such patients do badly with starch, and with sugar and other sweet articles. They should be put on a carefully selected diet consisting of the whole grains, which contain the gluten, silicates, starch, etc., conjoined, as bread made of whole wheat or bran, cracked wheat and oats; of carefully selected animal food, as meat, eggs and the like, and of green vegetables; acids may be allowed in moderation, as a little vinegar, or the acid fruits and vegetables, as the tomato; but if there is much irritation they had better be omitted. The amount of meat which is to be taken must be determined by the gastric capacity of the individual. We cannot lay down any hard and fast rules in regard to this. Some theories would indicate that this uric acid diathesis was well treated by giving a considerable amount of albuminoid substances and very little starch, but in practice you will find some patients who cannot digest a large amount of meat. In such cases you are obliged to rely greatly upon grains and skim-milk. Nearly always these patients require alkaline salines to be taken in connection with their meals. This is best done by the use of a mild saline (not purgative) water. No water has as high a reputation in the treatment of this disease as Vichy. There are, in this country, a number of springs of this type, but scarcely any of them are so free from irritating properties as Vichy. There is, at Saratoga, a so-called Vichy, which is about as good as any that we have in this country, but is not, I think, quite as well borne by the stomach as the original Vichy. We can substitute, for the alkaline water, the use of liquor potassæ or bicarbonate of sodium.

The use of an alterative diuretic should be associated with the alkaline water. The alterative diuretics which possess the greatest power in controlling irritability of the urinary passages are buchu, uva ursi and triticum, and probably in the order in which

I have mentioned them. A good plan is to order a glass of Vichy water to be taken one hour after each meal and with it a half drachm of the fluid extract of buchu, or one drachm of the fluid extract of uva ursi or triticum. This constitutes an excellent alterative and diuretic treatment for this class of cases; or, we may combine the fluid extract of one of these remedies with liquor potassæ or bicarbonate of potassium, giving, in addition, the compound syrup of sarsaparilla, or syrup of wild cherry bark, or other acceptable flavoring material.

Such patients nearly always require tonic treatment. You will usually find it necessary either to give iron alone, or, perhaps, more frequently, to give iron in combination with arsenic, belladonna and nux vomica. The iron may be given in the form of the pill of the carbonate, the citrate, the lactate, or the potassio-tartrate; thus, we may give a pill containing the following:

℞ Ferri et potassii tartratis, gr. jss,

Acidi arseniosi, gr. $\frac{1}{50}$ ad $\frac{1}{30}$,

Extracti belladonnæ, gr. $\frac{1}{12}$ ad $\frac{1}{6}$,

Extracti nucis vomicæ, gr. $\frac{1}{4}$.

M. et ft. pil. No. 1.

Signa: to be taken three times a day after meals.

Counter-irritation in some form is of great service, as is also the protection of the affected zone. A flannel belt, eighteen inches wide, extending from below the crest of the ilium to above the lower border of the ribs, made with plaits and whalebone, and worn day and night, is exceedingly useful in preventing the congestion of the kidney resulting from exposure.

Decided counter-irritation should be kept up over the spot. For this purpose I prefer the use of the actual cautery. It certainly powerfully modifies morbid action, and thus helps to prevent the attacks of pain; and as the touch of the hot iron is not nearly so painful as a single attack of pain, patients are usually anxious to have the applications repeated. Cantharides should not be used to blister, as it is apt to irritate the morbidly sensitive urinary tract, but if the actual cautery is not employed, antimonial ointment or dilute croton oil may be rubbed over the kidney.

Great care in hygiene is to be observed. Much exertion can not be indulged in without injury; exposure to cold and changes of the weather must be avoided.

In some cases, however, after everything has been done to relieve the patient, he goes on from bad to worse; he becomes more anæmic, the amount of pus increases, and œdema of the legs appears; the urine is more albuminous, showing that there is such a state of mal-nutrition and such a drain on the system as gradually to bring the patient lower and lower. With this, the attacks of pain are, perhaps, more frequent and severe, and on percussion the kidney may be found enlarged. In such a condition the question comes up as to what, if any, treatment can be resorted to in order to afford relief. All the means to which I have alluded have been employed, but something more is required.

We come then to the consideration of operative measures for the relief of this condition. It has been found that operations on the kidney were performed a long time ago, but these were only accidental, that is, a man was cut in the loins and a kidney protruded, and this was removed before it was discovered what it was. In some of these cases the patient has recovered. To Gustav Simon, of Heidelberg, however, undoubtedly belongs the credit of performing the first deliberate nephrotomy. In 1869, he removed a kidney from a woman with urethral fistula, and the patient recovered. Since Simon's operation there have been at least 100 cases of removal of the kidney. These have been collected by my friend, Dr. Robert P. Harris, of this city. His paper will be found in the *American Journal of Medical Sciences* for July, 1882. He publishes the results of 100 cases, a number of these operations (16) were for the removal of painful, wandering kidney. Martin, of Berlin, has operated on eight of these cases. This seems to be a common condition in

Austria, and Dr. Oser, of Vienna, goes so far as to say that one out of every ten women among the poor of Austria who have borne children has a kidney out of place, and that many of them cause much suffering. Other conditions, for which the operation has been performed, are cancer, tubercular disease, calculous pyelitis and calculous nephritis. The general results of these 100 cases may be stated to be that 50 per cent. died and 50 per cent. recovered. In many of the cases the examination of the organ showed that the disease was necessarily and inevitably fatal. It cannot be said that this result is entirely satisfactory. It is clear that we do not yet know the rules for the performance of this operation as well as we could wish. It is a somewhat curious fact that 90 out of these 100 operations were performed out of America, 21 having been performed in London alone. I have long desired that if a patient had a disease requiring this operation, he might come under my care; but during the past ten years I have not had a case where I considered the operation obligatory.

It has been performed both by abdominal section and by lumbar section. I should prefer the latter. The statistics are in favor of this method; in 46 cases, in which the lumbar operation was performed, 23 recovered and 23 died; in 50 cases of the abdominal operation, 27 recovered, 19 died and 4 were still under treatment.

In the case of this patient, none of us would recommend this operation; but in the case of a man with a family of young children, dependent on his labor for a livelihood, who, after being under careful treatment for one or two years showed a continued progress of the disease, and it became evident that his family would be beggared and he himself live in distress, deprived of all comfort by seeing those around him suffer from his condition, I should say that it was right to perform the operation, if he wished it. It is a satisfaction to know that after all attempts have been made and failed, there still remains a resort which, in cases of calculous pyelitis, gives good results in two out of every three cases; for the cases which brought up the mortality were cases of very serious disease, such as tuberculosis, carcinoma and large cysts. In two out of every three cases of renal calculus operated on, a cure has been effected; and further, we find that so far as we know at present (of course a longer observation may show that this is not so), the health of the patient does not seem to have been impaired by the loss of one of the kidneys.

This man will be placed on the hygienic and therapeutic treatment which I have suggested, and we shall have him report, from time to time, in order that its effect upon the spells of pain may be observed.

